# The Microvesicle Component of HIV-1 Inocula Modulates Dendritic Cell Infection and Maturation and Enhances Adhesion to and Activation of T Lymphocytes

**DOI:** 10.1371/journal.ppat.1003700

**Published:** 2013-10-17

**Authors:** Sarah K. Mercier, Heather Donaghy, Rachel A. Botting, Stuart G. Turville, Andrew N. Harman, Najla Nasr, Hong Ji, Ulrike Kusebauch, Luis Mendoza, David Shteynberg, Kerrie Sandgren, Richard J. Simpson, Robert L. Moritz, Anthony L. Cunningham

**Affiliations:** 1 Centre for Virus Research, Westmead Millennium Institute, Westmead, New South Wales, Australia; 2 University of Sydney, Sydney, New South Wales, Australia; 3 La Trobe Institute for Molecular Science, La Trobe University, Bundoora, Victoria, Australia; 4 Institute for Systems Biology, Seattle, Washington, United States of America; National Institute of Allergy and Infectious Diseases, National Institutes of Health, United States of America

## Abstract

HIV-1 is taken up by immature monocyte derived dendritic cells (iMDDCs) into tetraspanin rich caves from which the virus can either be transferred to T lymphocytes or enter into endosomes resulting in degradation. HIV-1 binding and fusion with the DC membrane results in low level *de novo* infection that can also be transferred to T lymphocytes at a later stage. We have previously reported that HIV-1 can induce partial maturation of iMDDCs at both stages of trafficking. Here we show that CD45^+^ microvesicles (MV) which contaminate purified HIV-1 inocula due to similar size and density, affect DC maturation, *de novo* HIV-1 infection and transfer to T lymphocytes. Comparing iMDDCs infected with CD45-depleted HIV-1_BaL_ or matched non-depleted preparations, the presence of CD45^+^ MVs was shown to enhance DC maturation and ICAM-1 (CD54) expression, which is involved in DC∶T lymphocyte interactions, while restricting HIV-1 infection of MDDCs. Furthermore, in the DC culture HIV-1 infected (p24^+^) MDDCs were more mature than bystander cells. Depletion of MVs from the HIV-1 inoculum markedly inhibited DC∶T lymphocyte clustering and the induction of alloproliferation as well as limiting HIV-1 transfer from DCs to T lymphocytes. The effects of MV depletion on these functions were reversed by the re-addition of purified MVs from activated but not non-activated SUPT1.CCR5-CL.30 or primary T cells. Analysis of the protein complement of these MVs and of these HIV-1 inocula before and after MV depletion showed that Heat Shock Proteins (HSPs) and nef were the likely DC maturation candidates. Recombinant HSP90α and β and nef all induced DC maturation and ICAM-1 expression, greater when combined. These results suggest that MVs contaminating HIV-1 released from infected T lymphocytes may be biologically important, especially in enhancing T cell activation, during uptake by DCs *in vitro* and *in vivo*, particularly as MVs have been detected in the circulation of HIV-1 infected subjects.

## Author Summary

Dendritic cells (DCs) are vital for immune recognition of pathogens as they capture, internalise, degrade and present foreign peptides to T lymphocytes. It is thought that HIV-1 hijacks the DCs functions, such as migration and maturation, to increase contact with the major target cell CD4^+^ T lymphocytes leading to dissemination throughout the body. Currently there is still some controversy over the ability of HIV-1 to infect and mature DCs, which may be due to differences in the inoculum used. Here we examined the effect of contaminating microvesicles (MVs) identified in HIV-1 preparations on HIV-1 modulation of DC function. We show that when MVs are present with HIV-1, the inoculum induces greater DC maturation and adhesion probably via cellular HSP90α and β and viral nef within the MVs. The functional consequences are reduced *de novo* replication of HIV-1 but increased clustering with T lymphocytes, resulting in increased T lymphocyte alloproliferation and HIV-1 transfer. As MVs are produced in HIV-1 susceptible cells and would be present *in vivo* due to HIV-1 induced cell death and hence are physiologically relevant, these results also indicate that MVs present in HIV-1 inocula should be considered when assessing HIV∶DC interactions.

## Introduction

Dendritic cells (DC), located throughout the body, but in particular in the male foreskin and the anogenital and cervical mucosa, are susceptible to HIV-1 infection [1]–[3]. They are also able to transfer HIV-1 to T lymphocytes, resulting in viral dissemination [4]. This ability to transfer HIV-1 to T lymphocytes is related to the function of DCs as antigen presenting cells, with the efficiency depending on the functional state of the cells. Immature DCs are highly endocytic; express low levels of major histocompatibility complex (MHC)-I and MHC-II complexes and co-stimulatory molecules but are considered to be poor processor and presenters to T lymphocytes. Mature DCs however are characterised by an up-regulation of surface expression of co-stimulatory molecules CD40, CD80 and CD86 and *de novo* expression of CD83 enabling efficient antigen presentation to and activation of T lymphocytes [5], [6]. Productive HIV-1 infection of T lymphocytes is much more efficient in activated cells than resting T lymphocytes [7], [8]. Therefore maturation of DCs following HIV-1 uptake is likely to be a key event in cell to cell spread. Whilst one group has found that HIV-1 does not induce maturation in either infected cells or uninfected bystanders [9], our group and others showed that HIV-1 induces a partial maturation of DCs [10]–[12]. Maturation genes were differentially expressed to a greater degree in cells treated with viable compared to non-viable virus indicating a role for replication over entry. Furthermore, this partial maturation was shown to be a result of p38 MAPK signalling [12]. The ability of DCs to form clusters with T lymphocytes and the subsequent formation of an immunological synapse is also important in HIV-1 transmission and is dependent on the interaction between ICAM-1 (CD54) on DCs and LFA-1 on CD4^+^ T lymphocytes [13] as evidenced by impaired DC mediated HIV-1 transmission to T lymphocytes in patients who lack LFA-1 on leukocytes [14]. Hence, the ability of HIV-1 to up-regulate the expression of adhesion and co-stimulatory molecules on DCs as part of their maturation may aid viral transfer to T lymphocytes and replication. HIV-1 transfer from DCs to T lymphocytes occurs within a viral synapse. Viral synapses are characterised by segregated supramolecular structures with a central cluster of envelope surrounded by a ring of ICAM-1 [15]–[17] to induce DC∶T lymphocyte adherence and allow for viral transfer.

Both immature and mature DCs are able to transfer HIV-1 to T lymphocytes by different mechanisms during 2 sequential phases of uptake, the first following vesicular uptake and the second after de novo infection of DCs transfer [18]–[21]. However mature DCs formed DC∶T lymphocyte conjugates more readily than immature DCs even in the absence of specific antigen [22]. An additional factor in HIV-1 transfer is the role of DC-derived microvesicle (MV)-associated HIV-1 particles. Protein-laden MVs are released from many different cell types, including the HIV-1 target cells T lymphocytes and DCs. A significant fraction of these vesicles are similar in size and density to HIV-1 virions, such that they sediment with HIV-1 even when vigorous methods to purify the virus are used [23]. In sucrose density gradient purified HIV inocula from PBMCs the ratio of virions to MVs is 1∶1–2 [24]. Vesicles and HIV-1 virions have similar, but not identical, protein compositions [23]–[27]. These vesicles are primarily shedding microvesicles (SMV) released from the cell surface, exosomes released from late endosomes/multivesicular bodies by exocytosis or apoptotic blebs (AB)s from dying cells, reviewed in [28]. The signalling molecule CD45 is expressed on all hemopoietic cells and is present in high concentrations on the MVs but specifically excluded from HIV-1 virions. It can therefore be used to purify viral stocks by removing contaminating vesicles [29]–[31]. Furthermore, the removal of vesicles by CD45-depletion is confirmed by the removal of other cellular proteins from the HIV-1 preparation, such as HLA-DR and actin [25].

In this study we have investigated the relative roles of HIV-1 and associated MVs on the maturation status of DCs and viral transfer to T lymphocytes using HIV-1 inocula containing or stripped of MVs. We have shown that MVs present within an HIV-1 inoculum enhance MDDC maturation which in turn induces more DC∶T lymphocyte clusters and results in higher levels of HIV-1 transfer to T lymphocytes. This induced maturation limits the productive infection of monocyte-derived DCs (MDDC). We propose that these MVs play a role in cell to cell spread of HIV-1 *in vitro* and possibly *in vivo*.

## Results

### Microvesicles Restrict HIV-1 Infection of MDDC

Previous work from our laboratory showed partial maturation of dendritic cells in response to HIV-1_BaL_, however we were unable to rule out the role of MVs in non-viral replication dependent DC maturation [10]. Therefore HIV-1_BaL_ viral stocks with or without the CD45^+^ MVs depleted were prepared as outlined by Bess et al [23]. The SUPT1-CCR5-CL.30 cell line was infected with VSV-G pseudotyped HIV-1p_BaL_, the CD45 contaminating MVs were removed with microbeads and the virus preparation was concentrated and purified further by centrifugation. The total HIV-1 p24 concentration as determined by ELISA was comparable for the HIV-1_BaL_(pellet) and HIV-1_BaL_(CD45^−^) viruses. Western blot analysis confirmed successful depletion of CD45 from the HIV-1_BaL_(CD45^−^) stock.

**Figure 1:**
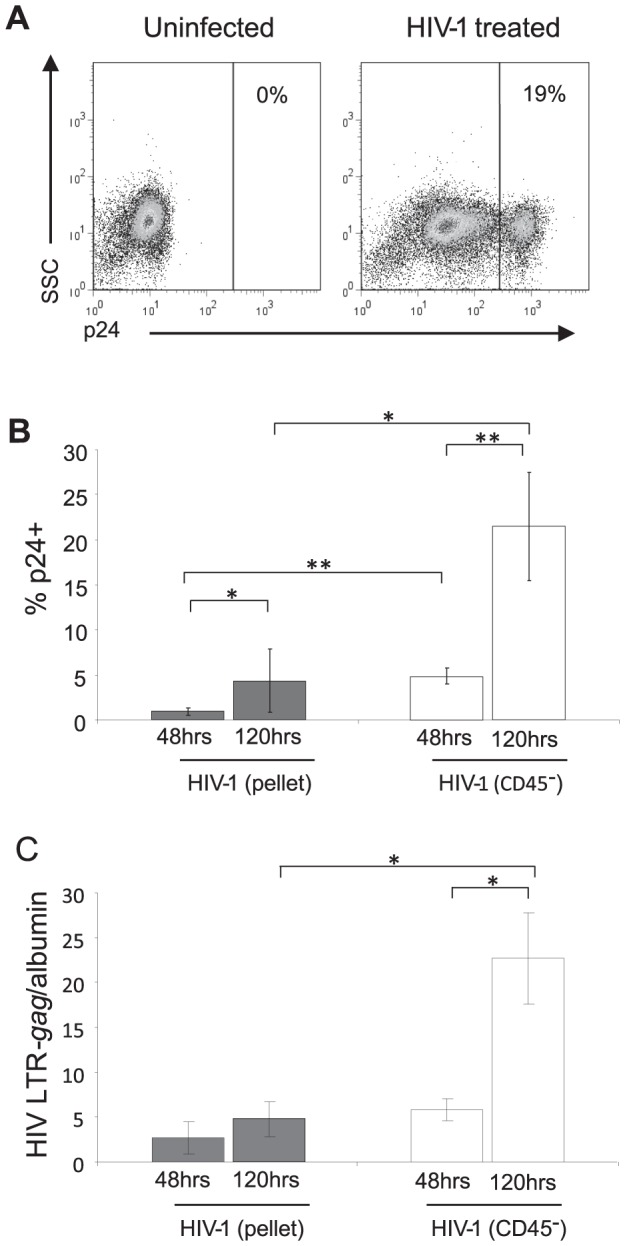
HIV-1_BaL_(CD45^−^) is more infectious in MDDCs than HIV-1_BaL_(pellet). MDDCs were infected for 48-1_BaL_(pellet) or HIV-1_BaL_(CD45^−^) at an MOI of 3 determined by TCID_50_, and assessed for intracellular HIV-1 p24 antibody by flow cytometry and HIV-1 proviral DNA by Q-PCR. A) Representative dot plots of untreated and HIV-1_BaL_(CD45^−^) exposed DCs identifying infected (p24^+^ cells in box on the right) and exposed but non-infected (p24^−^) DCs at 120 hours. B) The level of infection at 48 and 120 hours for both HIV-1_BaL_(pellet) and HIV-1_BaL_(CD45^−^) viruses by flow cytometry (mean +/− SEM, n = 12, *p<0.05 **p<0.01) and C) by Q-PCR (HIV-1 proviral DNA normalised by albumin values) (mean +/− SEM, n = 4, *p<0.05).

MDDCs were infected with either HIV-1_BaL_(CD45^−^) or HIV-1_BaL_(pellet) at a MOI of 3 measured as TCID_50_. Levels of productively infected cells were assessed by flow cytometry for intracellular HIV-1 p24 antigen at 48 and 120 hours post infection (Figure 1A and B). By 24 hours post infection, uptake of HIV-1 (p24 antigen) into vesicular/cave compartments of DCs have dissipated [32]. Depletion of CD45^+^ MVs increased the infection of MDDCs at both 48 and 120 hours (4.75+/−1.5% SEM, p<0.01 and 20.46+/−11% SEM, p<0.05 respectively). The level of HIV-1 proviral DNA was determined by Q-PCR and confirmed the flow cytometry infection values with increased infection of MDDCs at 120 hours when MVs were depleted (4.8+/−2% SEM at 48 hours and 22.7+/−5.1% SEM at 120 hours, p<0.05, Figure 1C). These results indicated that the CD45^+^ MVs limit the ability of the HIV-1 stock to productively infect DCs. This effect was not mediated via SAMHD1 as levels were similar in MDDCs treated with MVs from activated and non-activated CD4^+^ lymphocytes by western blot and densitometry (ratios of 0.9 and 0.9 respectively, compared to mock treated MDDCs; Figure S1)

### Removal of CD45^+^ Microvesicles Reduced the HIV-1 Induced Maturation of MDDCs

**Figure 2:**
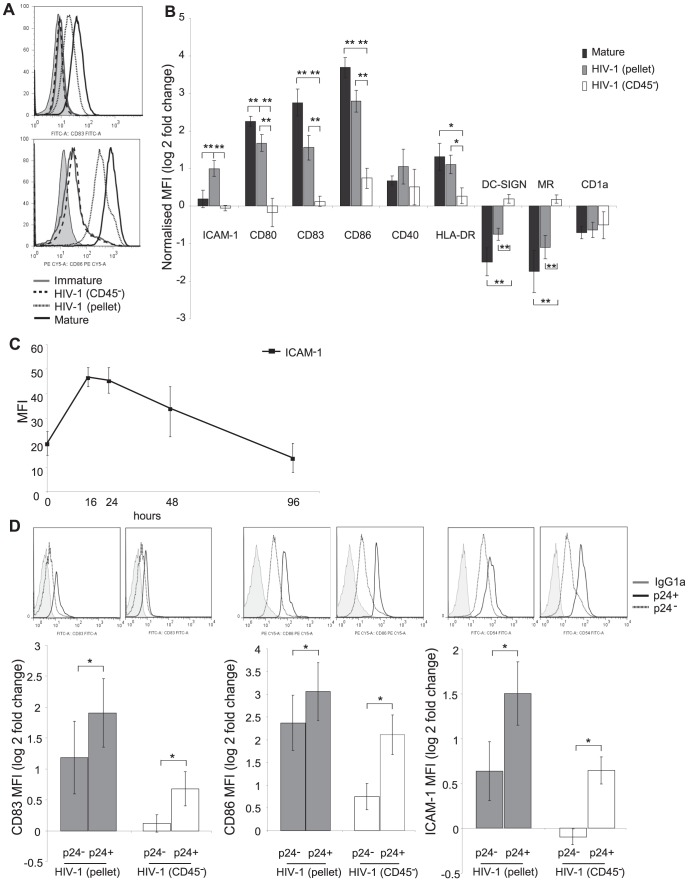
HIV-1_BaL_(CD45^−^) induces less maturation than HIV-1_BaL_(pellet). MDDCs were exposed to high titre HIV-1_BaL_(pellet), HIV-1_BaL_(CD45^−^) at a MOI of 3 or a potent maturation cocktail for 48 hours and expression of maturation determined by flow cytometry. A) Representative histograms for CD83 and CD86 shown (isotype grey tint, immature solid grey line, HIV-1_BaL_(CD45^−^) dashed line, HIV-1_BaL_(pellet) dotted line and mature solid black line). B) The levels of surface maturation marker expression with flow cytometry MFI results expressed as a ratio of normalised to untreated (mock/immature) controls as log (2) fold change (mean +/− SEM, n = 12, *p<0.05 **p<0.01). C) Kinetics over time of ICAM-1 expression determined by flow cytometry MFI for HIV-1_BaL_(pellet) treated MDDCs (mean +/− SEM, n = 3). D) Productively infected MDDCs are more mature than uninfected MDDCs for both virus stocks. Infected cells were identified by p24 antigen staining and the expression of CD83, CD86 and ICAM-1 was compared to that for the HIV-1 exposed uninfected cells. Representative histograms for the markers of p24^+^ (dashed line) and p24^−^ (dotted line) compared to isotype control (grey tint) for HIV-1_BaL_(pellet) and HIV-1_BaL_ (CD45^−^). Bar graphs delineating the expression of normalised maturation and co-stimulatory marker expressed as log (2) fold change to mock treated cells (mean +/− SEM, n = 5, *p<0.05).

The susceptibility of DCs to become productively infected with HIV-1 is related to the maturation status of the DCs [18]. We therefore hypothesised that the increased infection rate in MDDCs infected with HIV-1_BaL_(CD45^−^) virus was a consequence of lower maturation in these DCs compared to those infected with HIV-1_BaL_(pellet). In order to show this, DCs were infected with HIV-1_BaL_(pellet) or HIV-1_BaL_(CD45^−^) virus for 48 hours and the level of adhesion, co-stimulatory and other maturation markers was determined by flow cytometry and confirmed by q-PCR (data not shown). In agreement with our previously published work, HIV-1_BaL_(pellet) induced partial up-regulation of CD80, CD83, CD86 and HLA-DR as well as a partial down regulation of DC-SIGN and MR compared to a standard mixture of maturation inducing cytokines and prostaglandins (Figure 2A and B). In view of the importance of ICAM-1 in DC-T lymphocyte adhesion through viral synapses [33] its expression following HIV-1_BaL_(pellet) treatment was also examined and found to be elevated above that induced by the maturation mix. In contrast, the removal of the CD45^+^ MVs resulted in a failure to up-regulate surface expression of ICAM-1 and maturation markers on DCs including CD80, CD83, CD86 and HLA-DR, as well as a lack of down-regulation of CLRs compared to HIV-1_BaL_(pellet) treated cells (Figure 2B), indicating that MVs in the HIV-1 preparation induce some of the partial maturation.

To assess the kinetics of ICAM-1 up-regulation, the protein expression was determined at 0, 16, 24, 48 and 96 hours post infection by flow cytometry. We found that while ICAM-1 expression peaked at 16 hours post infection, the expression was still above baseline levels at 48 hours post infection (Figure 2C).

When the MDDC population was divided into the infected (p24^+^) and exposed but uninfected (p24^−^) populations it became apparent that within the HIV-1_BaL_(CD45^−^) treated cells, maturation was restricted to the p24^+^ productively infected cells and very little bystander maturation of p24^−^ cells was seen. This contrasted with the HIV-1_BaL_(pellet) treated DCs which showed increased CD83, CD86 and ICAM-1 expression in both infected and bystander cells (Figure 2D). Thus removal of MVs from HIV-1 preparations results in markedly reduced DC maturation and ICAM-1 expression.

### Addition of Microvesicles to HIV-1_BaL_(CD45^−^) Restores the Effects of HIV-1_BaL_(pellet)

**Figure 3:**
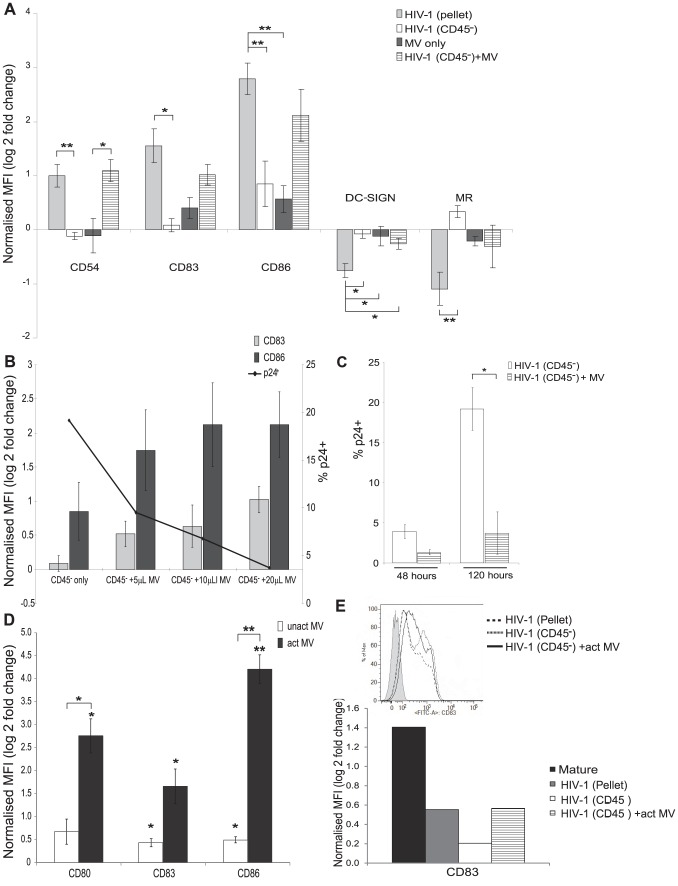
Addition of microvesicles to HIV-1_BaL_(CD45^−^) restores MDDC maturation. MDDCs were either treated with HIV-1_BaL_(pellet), HIV-1_BaL_(CD45^−^) alone, CD3/CD28 activated MVs (20 µl/3.5×10^5^ cells) alone or HIV-1_BaL_(CD45^−^) with the addition of CD3/CD28 activated MVs for 48 hours. To calculate the MV ‘inoculum’ the CD45 concentration of the MVs preparation was matched to the CD45 concentration of the HIV-1_BaL_(pellet) stock (see Table 1). A) The expression of maturation markers was determined by flow cytometry and compared to the maturation seen with the HIV-1_BaL_(pellet) stock (mean +/− SEM, n = 3, *p<0.05 **p<0.01). B) The expression of CD83 and CD86 was assessed and there was a positive correlation between amount of MVs added and the level of maturation seen in HIV-1 exposed MDDCs (mean +/− SEM, n = 3, CD83: p = 0.78, r = 0.9 and CD86: p = 0.27, r = 0.87). The level of p24^+^ cells was also measured by flow cytometry. C) The proportion of HIV-1 infected MDDCs at 120 hours was reduced by addition of MVs to the HIV-1_BaL_(CD45^−^) inoculum (mean +/− SEM, n = 3, *p<0.05). D) MVs from anti CD3/CD28 activated primary T lymphocytes also induce MDDC maturation. MDDCs (0.5×10^6^ cells/mL) were treated with MVs from CD3/CD28 activated or non-activated primary T lymphocytes at the same concentration as in panel A for 48 hours and expression of CD80, 83 and 86 measured by flow cytometry (mean +/−SEM, n = 3, *p<0.05, **p<0.01 compared to mock or, where bracketed, activated MVs are compared to non-activated MVs). E) HIV-1_BAL_(pellet) and MVs+HIV-1_BAL_(CD45^−^) also induce maturation of primary blood myeloid DCs. Blood myeloid BDCA1 DCs (0.5×10^6^ cells/mL) were isolated from blood and exposed to maturation mix, HIV-1_BAL_(pellet), HIV-1_BAL_(CD45^−^) and HIV-1_BAL_(CD45^−^)+MVs from activated SUPT1.CCR5-CL.30 cells at similar concentrations to panel A. CD83 expression was measured by flow cytometry. Representative histogram of three separate experiments.

To confirm that the differences induced by HIV-1_BaL_(pellet) compared to HIV-1_BaL_(CD45^−^) virus were a result of the MVs that were removed, we next devised a MV replacement experiment. The bead bound CD45^+^ MVs could not be used for these replacement assays as the CD45 microbeads caused DC maturation (data not shown). Additionally, it was not possible to remove the CD45 beads from the depleted fraction without damaging the MVs. We therefore generated MVs from SUPT1.CCR5-CL.30 cells used for propagation of HIV-1 stocks by stimulation with CD3 and CD28 antibodies for 3 days. The supernatant was subsequently concentrated as for HIV-1_BaL_ virus stock generation. This method of activating SUPT1.CCR5-CL.30 cells resulted in similar cytopathic phenotypic changes to those cells infected with HIV-1_BaL_. MVs from untreated SUPT1.CCR5-CL.30 cells had no effect on HIV-1 induced DC maturation or ICAM-1 expression (Figure 3A).

Once MV preparations were generated they were added back to the HIV-1_BaL_(CD45^−^) virus stock and then used to infect MDDCs. HIV-1_BaL_(pellet) was used as a positive control and the amount of MVs added was matched to the CD45 concentration of the HIV-1_BaL_(pellet) virus (determined by western blot densitometry, Table 1). In these experiments, the addition of isolated MVs to the HIV-1_BaL_(CD45^−^) virus resulted in increased expression of adhesion, co-stimulatory, maturation markers and CLRs on MDDCs while MVs alone did not induce maturation (Figure 3A). There was a concentration dependent effect (Figure 3B) as determined by CD83 and CD86 expression (r = 0.9 and r = 0.87 respectively).

**Table 1:** Western blot densitometry of HIV-1 preparations.

	HIV-1_BaL_ (pellet)	HIV-1_BaL_ (CD45^−^)	HIV-1_BaL_ (CD45^+^) bead
**Band intensity**	100+/−0.01	6.343+/−0.04	93.363+/−0.41

Net intensity normalised to HIV-1_BaL_ (pellet) +/− SE n = 3. Image J analysis.

In addition to the enhanced maturation, adding back MVs resulted in significantly less HIV-1 infection of MDDCs at 120 hours post infection (3.72+/−1.1% SEM, p = 0.05, Figure 3B and C). These results were further evidence that the maturation status of DCs and level of infection closely correlate (CD83 expression versus p24; r = −0.99).

In contrast to MVs from activated SUPT1.CCR5-CL30 cells, MVs from primary CD4^+^ lymphocytes activated by anti-CD3 and anti-CD28 antibodies induced significant maturation of MDDCs alone, without accompanying HIV-1_BaL_(CD45^−^) (Figure 3D).

In addition, exposure of primary blood myeloid BDCA1^+^ DCs to HIV-1_BaL_(pellet) showed enhanced maturation (up-regulated CD83) but this was diminished when they were exposed to HIV-1_BaL_(CD45^−^) and restored when MVs from activated SUPT1.CCR5-CL.30 cells were added to HIV-1_BaL_(CD45^−^) (Figure 3E).

### Clustering of HIV-1 Infected DCs with CD4^+^ Lymphocytes

**Figure 4:**
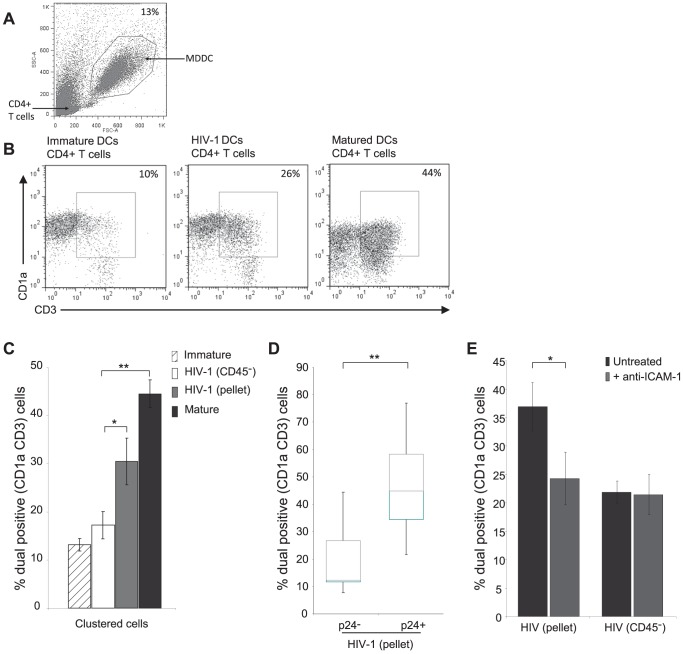
HIV-1 infection enhances clustering of DCs with autologous CD4+ T lymphocytes. MDDCs were either untreated or exposed to HIV-1_BaL_(pellet), HIV-1_BaL_(CD45^−^) (MOI 3) or a potent maturation cocktail for 48 hours and co-cultured with autologous CD4^+^ T lymphocytes at a ratio of 5 T lymphocytes∶1 DC for 45 minutes. A) Representative dot plot of the flow cytometry gating strategy for MDDC selection by FSC and SSC. B) Clusters were identified as double positive for CD3 (T lymphocyte marker) and CD1a (DC marker). Representative dot plots are shown. C) The percentage of DC clusters following treatment (mean +/− SEM, n = 10, *p<0.05 **p<0.01). D) DC:CD3^+^ cell clusters were identified in the p24^−^ and p24^+^ fractions of the co-culture and compared by box and whisker plots for the HIV-1_BaL_(pellet) virus (mean +/− SEM, n = 10, **p<0.01). E) Cluster formation following addition of neutralising antibody to ICAM-1 (mean +/− SEM, n = 3, *p<0.05).

ICAM-1 was markedly up-regulated in the MDDCs infected with HIV-1_BaL_(pellet) compared to maturation mix or HIV-1_BaL_(CD45^−^) virus (Figure 2B). This was further supported by investigating the expression in the infected populations and showing increased expression in p24^+^ MDDCs compared to p24^−^ MDDCs for both virus stocks (Figure 2D). The functional effects of increased ICAM-1 expression include increased and more stable DC∶T lymphocyte conjugates so we examined this by developing a DC∶T lymphocyte clustering assay using flow cytometry. Optimal conditions were obtained by co-culturing immature or mature MDDCs with autologous CD4^+^ T lymphocytes for 45 minutes at a ratio of 5 T lymphocytes∶1 DC. The cells were then labelled with CD1a (DC marker) and CD3 (T lymphocyte marker) antibodies. Within the DC population the proportion of cells that were also positive for CD3 were considered to be DC∶T lymphocyte clusters (gating strategy Figure 4A and B). This resulted in 44.5+/−2.9% SEM of mature DCs forming detectable clusters with T lymphocytes compared to 13.2+/−1.3% SEM of immature DCs forming clusters with T lymphocytes (Figure 4B). Results were validated by comparison between mature DCs involved in clusters by flow cytometry and by counting of those in contact with T lymphocytes using confocal microscopy (correlation >90%, data not shown).

DCs were then infected with either HIV-1_BaL_(pellet) or HIV-1_BaL_(CD45^−^) virus for 48 hours and co-cultured with autologous CD4^+^ T lymphocytes. The percentage of DCs that clustered with CD4^+^ T lymphocytes was significantly different between the two virus stocks (p<0.02). The HIV-1_BaL_(CD45^−^) treated DCs, which showed very little up-regulation of co-stimulatory markers or of ICAM-1, had levels of clustering with CD4^+^ lymphocytes (17.2+/−2.9% SEM) similar to the untreated negative control DCs. In contrast, the HIV-1_BaL_(pellet) treated DCs with higher ICAM-1 and co-stimulatory marker expression showed an increased cluster formation with CD4^+^ T lymphocytes (30.44+/−4.8% SEM), similar to mature DCs (Figure 4C).

After defining infected and bystander MDDCs separately, significantly higher clustering was seen in the p24^+^ DCs than in p24^−^ DCs for HIV-1_BaL_(pellet) (Figure 4D). Finally, blocking experiments with an antibody to ICAM-1 which reduced clustering to baseline levels, indicated that the elevated clustering seen with HIV-1_BaL_(pellet) treated MDDCs was due to ICAM-1 expression on DCs (Figure 4E). Thus, elevated ICAM-1 expression on HIV-1_BaL_(pellet) treated MDDCs results in greater DC∶T lymphocyte cluster formation than in HIV-1_BaL_(CD45^−^) treated MDDC.

### Microvesicles Enhance HIV-1 Infected DC Mediated Allogeneic Proliferation of T Lymphocytes

**Figure 5:**
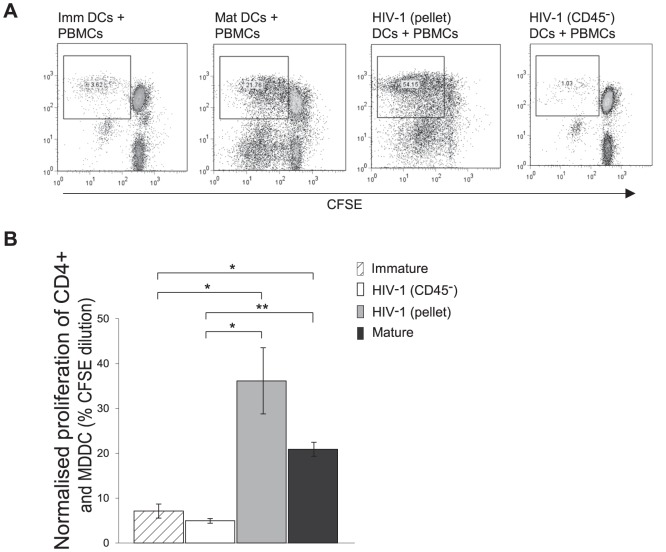
HIV-1 exposed MDDC stimulate PBMCs to proliferate. MDDCs exposed to HIV-1_BaL_(pellet) or HIV-1_BaL_(CD45^−^) for 48 hours were co-cultured with allogeneic PBMC at a ratio of 1 DC∶10 PBMC for 5 days. Proliferation was determined by CFSE dilution. The cells were stained for CD4^+^ T lymphocytes and analysed by flow cytometry. A) Representative dot plots of CD4^+^ T lymphocytes for each treatment are shown. B) The bar graph compares the proliferation between each treatment determined by adding the percentage proliferation normalised to live cell number of the CD4^+^ lymphocyte population and the MDDC population (mean +/− SEM, n = 3, *p = 0.05 **p = 0.01).

Next, the effects of differential DC maturation by the two HIV-1_BaL_ stocks upon T lymphocyte proliferation were examined. MDDCs were exposed to HIV-1_BaL_(pellet) or HIV-1_BaL_(CD45^−^) virus for 48 hours and then added to CFSE labelled allogeneic PBMCs from HIV-1 seronegative subjects at a ratio of 1 DC∶10 PBMC and co-cultured for a further 5 days. Following culture, cells were assessed for proliferation by flow cytometry. CD4^+^ T lymphocyte and MDDC populations were identified by size gating and the level of proliferation was assessed by CFSE dilution (Figure 5A). The percentage proliferation for each treatment was determined by normalising the CFSE dilution percentage to the percentage of live cells for both the PBMC and the MDDC populations. The values were then added to account for all CD4^+^ T lymphocytes present (Figure 5B). Immature and mature MDDCs co-cultured with CFSE labelled PBMC were used as negative and positive controls respectively (7.1+/−1.5% SEM, 10.7+/−0.9% SEM) (Figure 5B). HIV-1_BaL_(pellet) exposed DCs induced greater proliferation (36.2+/−7.2% SEM) of CD4^+^ T lymphocytes compared to mature DCs, whilst HIV-1_BaL_(CD45^−^) exposed DCs were significantly impaired in their capacity to induce T lymphocyte proliferation (4.98+/−0.5% SEM) compared to mature and HIV-1_BaL_(pellet) exposed DCs (p = 0.01 and p = 0.05 respectively).

### Microvesicles Enhance DC Mediated HIV-1 Transfer

**Figure 6:**
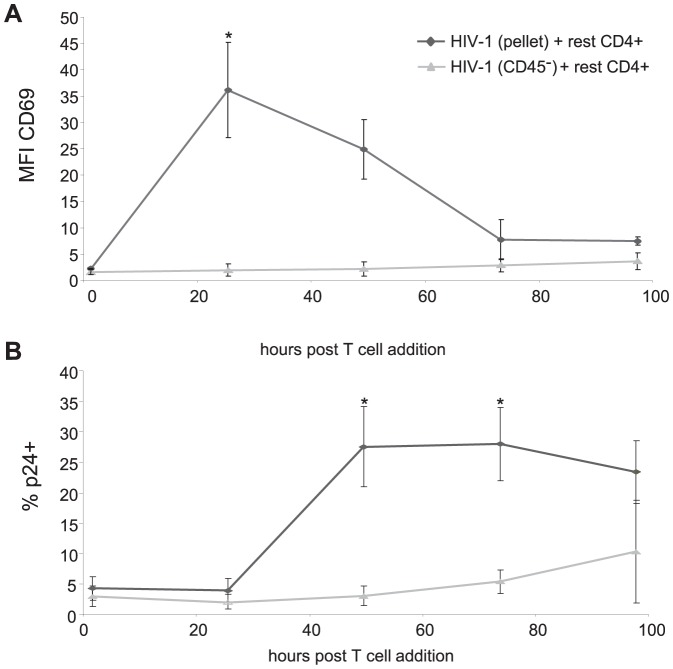
The presence of microvesicles enhances DC-stimulated CD4+ T lymphocyte activation and subsequent transfer of HIV-1. A) MDDCs treated with HIV-1_BaL_(pellet) or HIV-1_BaL_(CD45^−^) at (MOI 3) for 48 hours were co-cultured with unstimulated autologous CD4^+^ T lymphocytes. The expression of CD69 on T lymphocytes was determined by flow cytometry at 24, 48, 72 and 96 hours post T lymphocyte addition (mean +/− SEM, n = 3, *p<0.05). B) The level of infection of T lymphocytes was determined by gating on CD3+ cells (to exclude DCs) followed by p24 staining determined at 24, 48, 72 and 96 hours post T lymphocyte addition (mean +/− SEM, n = 3, *p<0.05).

As DCs are very efficient at transferring HIV-1 to T lymphocytes, we investigated the capacity of MDDC infected with the two different virus stocks to induce T lymphocyte activation and to transmit virus to T lymphocytes. MDDCs were infected with either HIV-1_BaL_(pellet) or HIV-1_BaL_(CD45^−^) virus for 48 hours and added to resting autologous CD4^+^ T lymphocytes for up to 96 hours. The levels of CD69 expression on CD4^+^ T lymphocytes, as a marker for T lymphocyte activation (Figure 6A), and the percentage of p24^+^ T lymphocytes (Figure 6B) was assessed over time. Consistent with their effects on DC maturation, only the HIV-1_BaL_(pellet) exposed DCs were able to induce activation of CD4^+^ T lymphocytes at 24 and 48 hours post co-culture.

CD4^+^ lymphocytes co-cultured with HIV-1_BaL_(pellet) exposed MDDCs resulted in higher and more rapid kinetics of infected CD4^+^ lymphocytes than when co-cultured with HIV-1_BaL_(CD45^−^) exposed MDDCs. Thus suggesting that DC maturation induced by MVs as well as virus in HIV-1_BaL_ preparations acts to enhance HIV-1 transmission.

### Characterisation of Microvesicles

**Figure 7:**
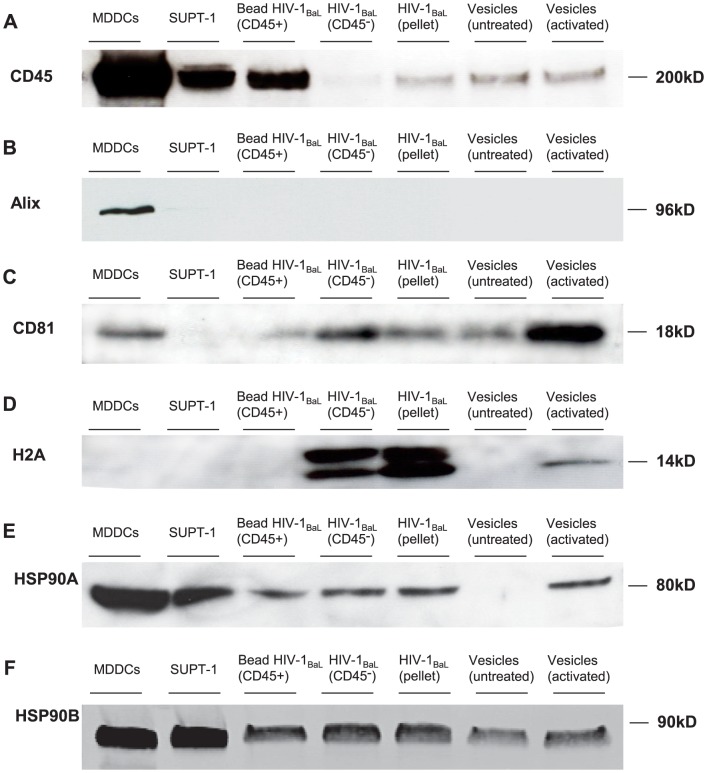
Western blot characterisation of HIV-1, microvesicles and parent cell line preparations. 30 µg of protein from MDDCs, SUPT1.CCR5-CL.30, bead bound HIV-1_BaL_(CD45^+^), HIV-1_BaL_(CD45^−^), HIV-1_BaL_(pellet), non-activated MV and activated MV preparations were compared for the expression of A) CD45, B) Alix, C) CD81, D) H2A, E) HSP90α F) HSP90β by western blot using SDS-page gel, nitrocellulose membrane and developed by chemiluminescence.

We next characterised the MVs in the HIV-1 and activated SUPT1.CCR5-CL.30 stocks. Initially, key candidate proteins present in MVs from the HIV-1_BaL_(pellet) and the MVs obtained from CD3/CD28 activated and non-activated SUPT1.CCR5-CL.30 cells were investigated by Western blot and compared to HIV-1_BaL_(CD45^−^), parent SUPT1.CCR5-CL.30 cells as well as the anti-CD45 bead bound HIV-1_BaL_ preparation (Figure 7 and Table 2). Both HIV-1 and MV preparations were positive for the tetraspanin/exosome marker CD81 but CD9 and Alix were undetectable. In addition, HIV-1_BaL_(CD45^−^), HIV-1_BaL_(pellet) and activated SUPT1.CCR5-CL.30 MVs contained the histone marker H2A indicative of ABs. Heat shock proteins (HSP) 90α and 90β (AB1) were also detected in both the viral and MV preparations (Figure 7).

**Table 2:** Western blot characterisation of HIV-1 and microvesicle preparations.

	MDDC	SUPT-1	Bead HIV-1_BaL_ (CD45+)	HIV-1_BaL_ (CD45−)	HIV-1_BaL_ (pellet)	Vesicles (activated)	Vesicles (non-act)
**CD45**	+++	++	++	−	+	+	+
**Alix**	+	−	−	−	−	−	−
**CD81**	+	−	+	+	+	++	+
**H2A**	−	−	−	++	++	+	−
**HSP90α**	++	++	+	+	+	+	−
**CD9**	+	++	−	−	−	−	−
**CD11a**	+	−	−	−	−	−	−
**CD40**	+++	+	−	−	−	−	−
**H3**	−	−	+	+	−	−	−
**CD40L**	+	+	+	+	+	−	−
**TNF-α** ψ	−	−	−	−	−	−	−
**HSP60**	+	+	−	−	−	−	−
**HSP70** ξ	−	−	−	−	−	−	−

+++ high protein expression, − below detection limit.

ψ positive in PBMCs,

ξ positive in transfected lysate.

To fully characterize the protein complement of the MVs from the two HIV-1 inocula and also the anti-CD45 bead bound material compared to those from activated and non-activated SUPT1.CCR5-CL.30 cells we separated the protein bands by gel electrophoresis and subjected them to Tandem Mass Spectrometry. After filtering for non-human contaminating proteins present in the fetal calf serum, 266 proteins with >0.01% share of the total were detected in HIV-1_BaL_(pellet), 255 in HIV-1_BaL_(CD45^−^), 274 in the anti-CD45 bead bound preparation, 462 in MVs from activated SUPT1.CCR5-CL.30 and 143 from non-activated SUPT1.CCR5-CL.30 MVs. The key proteins characteristic of MVs which were identified are shown in Tables 3 and 4. These included α-enolase, pyruvate kinase, actin and actin related proteins, the tetraspanins, CD9 and CD81, and several heat shock proteins especially HSP90α and β. HSP90α and HSP90β, were the only cellular proteins present in concentrations of >0.01% which were likely to act as DC maturation stimuli in HIV-1_BaL_(pellet) and activated SUPT1.CCR5-CL.30 MVs and were present at high concentrations in HIV-1_BaL_(pellet) (0.32% and 0.20% of peptide spectrum IDs respectively) and in the activated SUPT1.CCR5-CL.30 MVs (0.40% and 0.25% respectively) (Table 4). They were also in the top 20 of the 200–400 identified proteins. HSP90α and β were present at 3 fold and 6 fold higher concentrations respectively in MVs from activated than in non-activated uninfected SUPT1.CCR5-CL.30 cells, By mass spectrometry there was little difference in HSP90α concentrations in HIV-1_BaL_(pellet) and HIV-1_BaL_(CD45^−^) but HSP90β appeared to be present at lower levels in HIV-1_BaL_(pellet) (0.20%) than after depletion of MVs with CD45 antibody bound beads (0.63%) (Table 4), although this was not supported by western blot and densitometry (Figure 7). Here both HSP90α and β were evenly distributed between HIV-1_BaL_(pellet) and HIV-1_BaL_(CD45^−^) inocula and both were present on CD45+ beads. However both were markedly increased, especially HSP90α in MVs from activated verses non-activated SupT1 cells. HSP70/71 were present at very low concentrations in all preparations (by MS).

**Table 3:** Characteristic microvesicle specific proteins present in anti-CD45 bead bound HIV-1 inoculum and SUPT1.CCR5-CL.30 derived microvesicles (expressed as % share of spectrum IDs).

	Bead HIV-1_BaL_ (CD45+)	Vesicles (activated)	Vesicles (non-activated)
**α-enolase**	0.95	0.569	0.149
**Pyruvate kinase**	0.716	0.28	0.029
**CD81**	0.072	0.071	0.032
**CD9**	0.052	0.052	0.032
**Actin/related proteins**	0.39	0.063	0.028

**Table 4:** Concentrations of heat shock proteins and HIV-1 Nef and Gag in HIV-1 inocula and of HSPs in uninfected SUPT1.CCR5-CL.30 derived microvesicles (MVs).

	HIV-1_BaL_ (pellet)	HIV-1_BaL_ (CD45−)	MVs (activated)	MVs (non-activated)
**HSP90α**	0.32	0.33	0.40	0.13
**HSP90β**	0.20	0.63	0.25	0.04
**HSP70/71**	0.03	0.00	0.00	0.00
**HIV-1 nef**	0.023	0.00		
**HIV-1 Gag**	2.17	3.82		
**HIV-1 env**	0.021	0.073		

The HIV-1 accessory protein nef was present in HIV-1_BaL_(pellet) (at lower levels than gag or env), but completed depleted from HIV-1_BaL_(CD45^−^) virus. However, Gag/pol and env peptides were concentrated 1.5 to 3 fold in HIV-1_BaL_(CD45^−^) compared to HIV-1_BaL_(pellet) (Table 4). Direct comparison of the levels of HIV-1 proteins concentrated on the anti-CD45 beads showed a rank order of gag/pol (mean 2.16% share), env (mean 0.12%), nef (mean 0.033%), vpr (0.015%).

**Figure 8:**
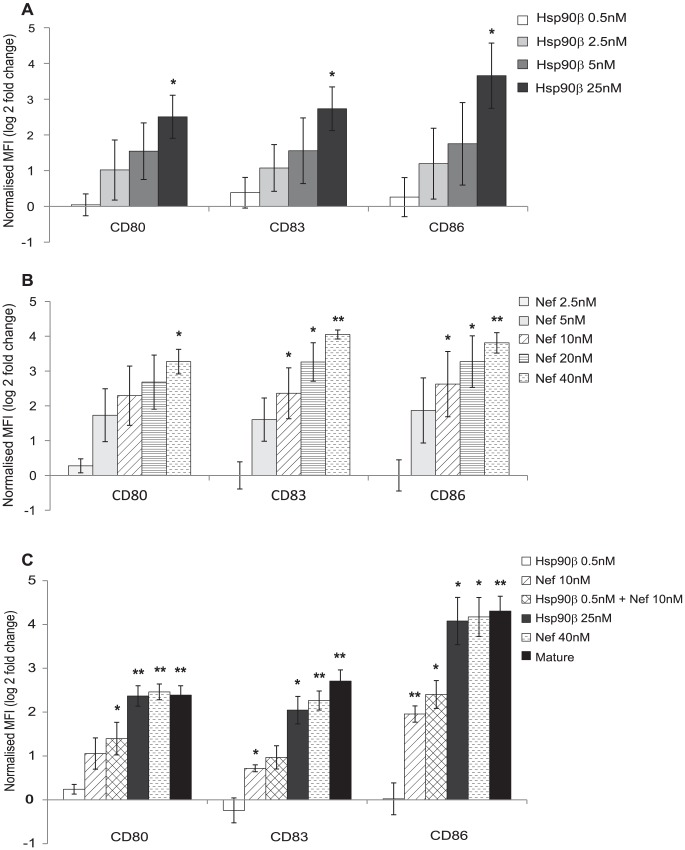
Recombinant HSP90β and recombinant Nef induce MDDC maturation and ICAM-1 expression. MDDCs (0.5×10^6^ cells/mL) were treated with recombinant proteins, A) HSP90β and B) nef at variable concentrations for 48 hours. The effects on the maturation markers, CD80, CD83 and CD86, were measured by flow cytometry and expressed as log (2) fold change in MFI compared to mock treated MDDCs (mean +/− SEM, n = 3, *p<0.05, **p<0.01). C) MDDCs (0.5×10^6^ cells/mL) were treated with recombinant nef at 10 nM (lowest significant effective concentration) and recombinant HSP90β at 0.5 nM (completely ineffective concentration on MDDC maturation alone) or maturation mix for 48 hours and expression of CD80, CD83 and CD86 measured by flow cytometry and expressed as log (2) fold change in MFI compared to mock treatment (mean +/− SEM, n = 3, *p<0.05, **p<0.001).

To directly examine the effect of HSPs 90α and β and nef on MDDC maturation, graded concentrations of recombinant HSP 90α (Figure S2) and 90β and of nef were added to MDDCs (Figure 8A and B). Each alone induced maturation and ICAM-1 expression in a concentration dependent fashion. HSP90β was more potent than HSP90α (minimal effective concentrations 2.5–5 nM, significant at 25 nM for HSP90β and for HSP 90α effective at 25 nM, significant at 50 nM). The lowest effective concentration of nef was 10 nM (Figure 8B).

The combination of recombinant nef at its lowest effective concentration (10 nM) with HSP90β at a concentration (0.5 nM) tenfold below its minimum effective concentration (5 nM) enhanced maturation above nef alone, especially for CD80 (Figure 8C). Thus, MVs contain viral and cellular proteins that are capable of potent stimulation of DC maturation and ICAM-1 expression.

## Discussion

Purification of HIV-1 preparations released from infected T lymphocytes by removal of the contaminating MVs can be achieved by CD45 depletion [23], [25], [30]. In this paper we have described experiments using HIV-1 inocula with and without depletion of CD45^+^ MVs and then restoration of MVs from activated uninfected SUPT1.CCR5-CL.30 or primary T cells to investigate the biological role of MVs in HIV-1 uptake by and infection of MDDCs. When compared, we observed that HIV-1_BaL_ depleted of MVs infected DCs to a greater extent than HIV-1_BaL_ containing MVs. However, HIV-1 depleted of MVs induced much less MDDC maturation, ICAM-1 expression and clustering with CD4^+^ T lymphocytes than HIV-1 containing MVs. This should also be applicable in the tissue microenvironment in vivo.

Several HIV-1 restriction factors have been described for DCs, including the constitutively expressed APOBEC3G [34], SAMHD1 [35] and the HIV induced interferon stimulated genes [36]. Our finding that MVs within the viral inocula restrict HIV-1 infection of DCs may reflect a further mechanism by which DCs are able to control viral infection, or more importantly, their induction of partial maturation may facilitate viral transfer to CD4^+^ lymphocytes. It has been well documented that immature MDDCs are more HIV-1 susceptible than mature MDDCs [37]–[40] due to differences in CCR5 expression which is required for HIV-1 fusion [41] as well as reverse transcription and/or post-integration blocks [42], [43]. The presence of MVs in the inoculums resulted in increased maturation (but not SAMHD1 levels) of both productively infected and bystander DCs. This enhanced maturation, particularly in the bystander cells may explain the lower levels of HIV-1 infection in DCs infected with HIV-1_BaL_(pellet).

In this study we show that productively infected DCs are more mature than exposed bystander cells, confirming our previous findings that the maturation was a result of HIV-1 replication, as well as exposure [10], [11]. The role of HIV-1 replication in DC maturation was strengthened by the observation that HIV-1_BaL_(CD45^−^) productively infected DCs exhibited some maturation. MVs present in the HIV-1_BaL_(pellet) preparation must have a role in uninfected bystander DC maturation as their removal eliminated this effect. The greater maturation seen in the productively infected DCs with HIV-1_BaL_(pellet) compared to HIV-1_BaL_(CD45^−^) suggested an additive effect from the MVs. The use of two different HIV-1_BaL_ inocula may contribute to an understanding of the different routes of entry of HIV-1 into DC. At early time points post infection HIV-1 and MVs induce maturation of infected DC as well as uninfected bystander DC due to vesicular uptake (into ‘caves’ and late endosomes). At later time points (>48 hpi) however, the effects of replicating HIV-1 on DC maturation may dominate. It is of note that we have assumed in this paper that HIV-1 p24^−^ DCs were uninfected. This is probably true of the majority of DCs, although a small proportion of these cells will be in the early stages of infection but do not yet express HIV-1 DNA^+^ p24 antigen at detectable levels. Furthermore while CD14^+^ monocytes are commonly used as a source of *in vitro* MDDCs, the use of MDDCs in this study is strengthened by the observations that *in vivo* equivalent cells in both mice [44], [45] and recently in humans can be generated during physiological stress [46], [47]. Nevertheless we also found similar effects of HIV-1 inocula with and without MVs on primary blood BDCA1^+^ myeloid DCs.

The adhesion molecule ICAM-1 was investigated due to its role in immunological and viral synapse formation [48] and was shown to be expressed to a greater extent than other maturation and co-stimulatory markers examined by HIV-1_BaL_(pellet) compared to matured DCs. This supports the findings that DC subsets expressing higher levels of ICAM-1 transmit HIV-1 more efficiently [49]. The functional consequences of DC maturation and ICAM-1 expression were assessed using a novel flow cytometry based assay to assess DC∶T lymphocyte clustering interactions. The results showed that HIV-1_BaL_(pellet) treated DCs cluster with T lymphocytes to a greater extent than with immature DCs although less than matured DCs. Furthermore productively infected cells cluster more than uninfected bystander DCs which were exposed to the inoculum. Previous work has shown that HIV-1 viral protein nef increases the capacity of DCs to form clusters with allogeneic CD4^+^ T lymphocytes which increased immunological synapse formation [50], [51] and blocking ICAM-1 has been shown to decrease HIV-1 transfer [13], [14]

As well as different levels of DC maturation seen when cells were treated with HIV-1_BaL_(pellet) or HIV-1_BaL_(CD45^−^), corresponding rates of T lymphocyte proliferation and HIV-1 transfer were observed. DCs treated with HIV-1_BaL_(pellet) led to greater CD4^+^ and CD8^+^ T lymphocyte proliferation than those treated with HIV-1_BaL_(CD45^−^). In addition, HIV-1_BaL_(pellet) led to transfer of HIV-1 to both activated and resting CD4^+^ T lymphocytes, while HIV-1_BaL_(CD45^−^) was unable to activate contacting CD4^+^ T lymphocytes and was only able to be transferred to activated CD4^+^ T lymphocytes, probably due to decreased ICAM-1 expression and clustering. This indicated a significant role for infected and especially bystander DC maturation due to presence of MVs, on HIV-1 transfer. These results together are important as, until now, it has not been possible to determine the individual roles of maturation and infection on the spread of HIV-1 from DCs to T lymphocytes.

The viral inoculum *in vivo* (e.g. in semen or blood) is derived from lysed infected CD4^+^ lymphocytes and therefore likely to contain MVs, so this is a more physiologically relevant viral inoculum. HIV-1 infected activated T lymphocytes burst release HIV-1 [52], [53] which also releases cell debris and MVs. Such MVs containing HIV-nef have been identified and quantified in vivo in (ultracentrifuged) plasma from HIV infected [54], [55].

**Figure 9:**
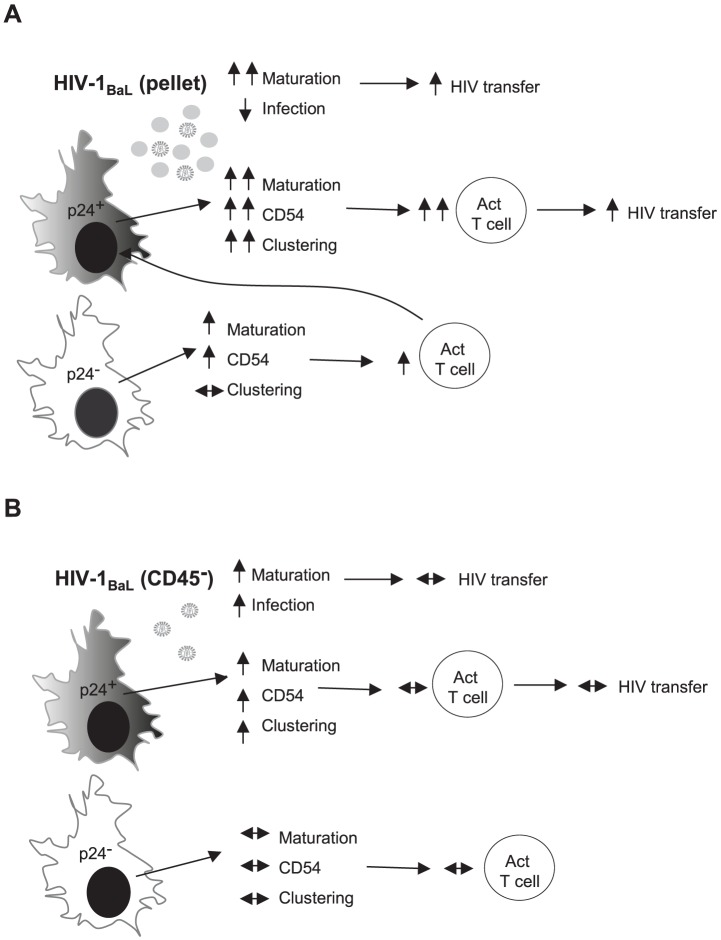
A model of HIV-1 induced and bystander effects on MDDCs. Diagram of MDDCs infected with either A) HIV-1_BaL_(pellet) or B) HIV-1_BaL_(CD45^−^) virus. MDDCs exposed to HIV-1_BaL_(pellet) had increased maturation, limited infection and when co-cultured with T lymphocytes, T lymphocyte activation and HIV-1 transfer was observed. When the MDDCs were assessed based on their p24 status to delineate between infected (p24^+^) and uninfected bystanders (p24^−^), there were notable differences with infected MDDCs showing greater maturation, ICAM-1 expression and clustering. However, as only 1–2% of the population were considered infected, T lymphocytes activated by bystander MDDC interactions could also then interact with the small infected population resulting in HIV-1 transfer to these cells. In contrast, MDDCs exposed to the HIV-1_BaL_(CD45^−^) had limited MDDC maturation and increased HIV-1 infection. The infected MDDCs did show some maturation, ICAM-1 expression and clustering to T lymphocytes, whereas the bystander cells did not. When co-cultured with T lymphocytes, little T lymphocyte activation and HIV-1 transfer was observed, possibly due to a small number of exposed MDDCs able to activate the T lymphocytes (5–10%) compared to the majority of MDDCs when exposed to HIV-1_BaL_(pellet). The differences seen between the two virus stocks were a due to the presence of MV in the HIV-1_BaL_(pellet) virus. up arrow = up-regulated compared to mock, down arrow = down-regulated compared to mock and left-right arrow = no change compared to mock.

Our results show that *in vitro* a low proportion of HIV-1 infected DCs, if accompanied by maturation, is more important in virus transfer to T lymphocytes than high levels of DC infection without maturation (Figure 9). Brenchley *et al* described the systemic activation of the immune system in relation to HIV-1 immunopathology [56] whereby CD4^+^ T lymphocytes are constantly activated by leaky gut products which support HIV-1 replication and lead to significant CD4^+^ T lymphocyte death [57]. It was shown that circulating LPS was significantly increased in chronically HIV-1 infected individuals and in simian immunodeficiency virus (SIV)-infected rhesus macaques, while this was not seen in non-pathogenic SIV infection of sooty mangabeys [58]. By analogy MVs released in parallel with HIV-1 should also induce DC activation of CD4^+^ T lymphocytes *in vivo* and this may contribute to HIV-1 replication and spread.

Recent studies have shown that many virally infected cells secrete MVs, including vaccinia virus [59], hepatitis C [60] and Epstein Barr virus (EBV) [61], [62]. The analysis of the protein complement of the MVs present in the anti-CD45 bead bound and activated SUPT1.CCR5-CL.30 preparations, especially by Mass Spectrometry, detected candidate maturation inducing proteins: the HSPs 90α and β, and HSPs 70/71 (the latter at very low levels) and the HIV-1 protein nef [63].HSPs function as chaperone proteins with varied cellular locations (see review: [64]) and have been shown to act as a source of antigen and may play a role in the transfer of peptides to APCs when released into the extracellular milieu in exosomes [65], [66]. When expressed on the cell-surface HSPs are capable of activating the immune system *in vivo* [67], [68]. Several heat shock proteins (HSPs 60, 70/71, 96) have been reported to induce maturation or activation of DCs to variable extents, independently of lipopolysaccharide,, via NFκB and the MAPK pathway [63], [69]–[73]. Here we have shown that HSPs 90α and β, are the major cellular HSPs expressed in MVs and are fairly evenly distributed between MVs and MV depleted HIV. Both purified recombinant HSP90α and β induce maturation of MDDCs alone with HSP90β being the most potent.

Nef was also detected in MVs but depleted in the MV stripped HIV inoculum. Pure reombinant nef was a potent inducer of MDDC maturation. Nef has previously been shown to be secreted in exosomes and induce bystander cell effects, [74] and to be taken up by DCs and induce IL-12 secretion and clustering with T lymphocytes. Effects on DC maturation and ICAM-1 expression were not studied [51]. In our experiments a combination of both proteins induced higher levels of DC maturation/ICAM-1 expression together than either alone. Nevertheless MVs from uninfected activated SUPT1.CCR5-CL.30 cells or primary T lymphocytes containing only HSP90α/β but at high concentrations, could substitute for the MVs from infected cells, containing both HSP90α/β, and nef. Thus both HSP90α/β are responsible for the effect MVs derived from uninfected activated primary T and SUPT1.CCR5-CL.30 cells in enhancing maturation of MDDCs.

The complexity of the HIV-1 and MV effects on DCs is dissected into 4 scenarios in panels A and B of Figure 9. In panel A the whole MDDC culture is exposed to an inoculum of HIV virions, containing HSP90α/β, and MVs, containing HSP90α/β and nef, leading to maturation of both infected and uninfected bystander DCs (which are the majority). Additionally infected DCs produce endogenous nef, possibly explaining the higher degree of maturation. In panel B the whole MDDC culture is exposed to HIV containing (a lower dose of) HSP90α/β but not nef, which is insufficient to induce maturation in bystander DCs. Exogenous MVs from activated uninfected T lymphocytes, bearing HSP90α/β, are sufficient to restore the maturing effect of the inoculum. In the infected MDDCs the production of endogenous nef in the infected DCs probably combines with exogenous HSP90α/β, to induce a low degree of maturation. These proteins may induce signalling either through the Vav pathway (for nef) or plasma membrane TLR2/4 (for HSPs) [51].

In conclusion, *in vitro* DC maturation and ICAM-1 up-regulation is induced by MVs and HIV-1 exposure in all cells of the culture and by HIV-1 replication as well as exposure to MVs in infected DCs. In turn this maturation and adhesion molecule up-regulation induces greater cluster formation and activation of CD4^+^ T lymphocytes and subsequent transfer of HIV-1. Hence the biological role of MVs in HIV-1 emerging from lysing CD4^+^ lymphocytes needs to be considered during DC infection *in vitro* and *in vivo*. Activation of T lymphocytes *in vivo* may contribute to the progressive immunodeficiency of HIV disease. Conversely exposure of uninfected bystander DCs in vivo could lead to endosomal then cytosolic uptake of MVs containing nef and HSP90α/β, maturation of DCs and induction of CD8 lymphocyte responses to nef, which is disproportionately recognised as a target HIV protein.

## Materials and Methods

### Cell Culture

MDDCs were generated from CD14^+^ monocytes isolated from peripheral blood mononuclear cells (PBMC) of anonymous blood donors from the Australian Red Cross Blood Service, Sydney using CD14 magnetic beads (Miltenyi Biotech; Gladbach, Germany) as described previously [10].

When required, MDDC were matured for 48 hours in maturation mix consisting of (v/v) final concentration 50 pg/mL IL-1β (R&D Systems; Minneapolis, MN, USA), 5 U/mL IL-6 (R&D Systems), 50 pg/mL TNF-α (R&D Systems) and 5 ng/mL PGE2 (Sigma; Milwaukee, WI, USA) in 0.1% (w/v) bovine serum albumin (BSA, Sigma) PBS.

CD4^+^ T lymphocyte isolation (97% purity) was performed using magnetic beads (Miltenyi Biotech) according to the manufacturer's protocol.

Primary blood myeloid DCs were isolated from PBMCs collected from whole blood, using a negative selection magnetic bead myeloid DC isolation kit (Miltenyi), as per manufacturer's instructions. Isolated mDCs were cultured at 1×10^6^ cells/mL in RF10 (RPMI supplemented with 10% FCS and no cytokines) and collected at 24 h post treatment.

Phenotype/purity was checked by Flow cytometry. Stained with Live/Dead Aqua (Invitrogen), Lin marker (BD), HLA-DR (Biolegend), BDCA-1 (Miltenyi) and BDCA-3 (Miltenyi). Maturation was checked by staining with Live/Dead, HLA-DR, BDCA3, CD83 (BD) and CD86 (BD). Cells were gated by size, Live, and HLA-DR^+^ before gating on BDCA-3^+^ and BDCA-3^−^ (“BDCA-1”). CD83 and CD86 expression was analysed on the separate BDCA-1/BDCA-3 populations.

### Recombinant Proteins

Recombinant HSP90α and β free of LPS, were purchased from Abcam, UK (at 2.7 mg/mL and 1.7 mg/mL respectively) and recombinant nef from BioAcademia Inc. Japan (at 0.48 mg/mL)

### Virus and Microvesicle Preparations

HEK293T cells (Human Embryonic Kidney cells, NIH AIDS Research and Reference Reagent Program) were transfected with pWT/_BaL_ (NIH AIDS Research and Reference Reagent Program, contributed by Dr. Bryan R. Cullen) and pHEF-VsV-g (NIH AIDS Research and Reference Reagent Program, contributed by Dr. Lung-Ji Chang) plasmids using polyethylenimine (PEI, Polyscience; Warrington, PA, USA) to generate VsV-g pseudotyped p_BaL_. Purified high titre HIV-1_BaL_ stocks in the order of 2×10^9^ TCID_50_/mL were generated by infection of SUPT1.CCR5-CL.30 cells (Human Non-Hodgkin's T lymphocyte Lymphoma, contributed by Prof. James Hoxie at the University of PA) with VsV-g pseudotyped p_BaL_. HIV-1 infected supernatants were concentrated using tangential filter concentration using the Millipore Lab scale system (Millipore; Billerica, MA, USA) and 2× Pellicon filters connected in parallel (300 kDa) (Millipore). When required, CD45^+^ MVs were depleted from supernatant using CD45 magnetic beads (Miltenyi Biotech). Virus (18 mLs) was incubated at room temperature with 2 mLs microbeads for 2 hours before adding to the top of a LS column. CD45 depleted virus that flowed through the column as well as non-depleted supernatants were concentrated further by ultracentrifugation with 1 mL under-layed 20% sucrose cushion and centrifuged at 100,000×g (Beckman Optima XL-100K Ultracentrifuge with 70Ti rotor) at 4°C for 90 minutes. Virus content was determined by p24 gag ELISA as per manufacturer's instructions (Beckman-Coulter; Hialeah, FL). The 50% tissue culture infectious dose (TCID_50_) values were generated in TZM-BL cells (NIH AIDS Research and Reference Reagent Program, contributed by John Kappes and Xiaoyun Wu) measured by LTR β-galactosidase reporter gene expression after a single round of infection [75].

MVs from activated SUPT1.CCR5-CL.30 or primary T lymphocytes were generated using antibodies to CD3 (1 µg/mL, BD Pharmingen; Becton Dickinson; San Jose, CA) and CD28 (5 µg/mL, BD Pharmingen) added to 100×10^6^ cells, cultured for 3 or 6 days respectively alongside unstimulated cells and the supernatant concentrated as above. The CD45 concentration for virus and MV was determined by western blot. Endotoxin levels for all virus and MV stocks were negative using the ToxinSensor Chromogenic LAL Endotoxin Assay Kit (GeneScript; Piscataway, NJ).

### MDDC Treatment with HIV-1, Microvesicles or Recombinant Proteins

Immature day 5 MDDCs were infected with pelleted (HIV-1_BaL_(pellet)) or CD45depleted (HIV-1_BaL_(CD45^−^)) virus in 200 µL media with a MOI of 3 at 37°C for 2 hours before resuspending at 1×10^6^/mL and incubating further as required. MVs were added at 20 µL/mL to match the CD45 concentration to HIV-1_BaL_(pellet) virus stocks. Recombinant proteins were added at various final concentrations to MDDCs cultured at 0.5×10^6^cells/mL in RF10+cytokines. Harvested cells at 48 hours post treatment for FACS analysis.

### Flow Cytometry

Direct conjugated mAbs directed against ICAM-1-fluorescein isothiocyanate (FITC) (Beckman Coulter), CD80-Phycoerythrin-Cy5 (PE-Cy5), CD83-FITC, CD86-PE-Cy5, HLA-DR-Allophycocyanin (APC), MR-APC, CD1a-FITC, CD40-FITC, CD3-APC, CD8-PE-Cy5 and CD69-APC (BD Pharmingen) and CD4-PE (Sigma) were used for surface staining. Antibody staining was performed at 4°C for 30 minutes using fluorescence-activated cell sorter buffer (1% (v/v) Human Ab Serum, 2 mM EDTA and 0.1% (w/v) sodium azide made up in PBS). For intracellular staining HIV-1_BaL_ or mock treated MDDCs were fixed and permeabilised in Cytofix/Cytoperm (BD), re-suspended in permwash buffer (1% (v/v) human AB serum (Sigma), 0.1% (w/v) saponin, 0.1% (w/v) sodium azide, made up in PBS at 4°C) and incubated for 2 hours with PE conjugated p24 (clone KC57-RD1, Beckman Coulter; Fullerton, CA). IgG isotype control antibodies were incubated with cells to control for nonspecific binding. Cells were then analysed with a FACS-Canto flow cytometer (Becton Dickinson) and FlowJo (Tree Star Inc., Ashland, OR).

Blocking of ICAM-1 (clone HA58, 5 µg/2×10^5^ cells, BD Pharmingen) was performed at 37°C for 30 minutes.

### Q-PCR

2×10^5^ cells were lysed at 60°C for 90 minutes in Q-PCR Lysis Buffer (10 mM Tris-Hydrochloride, 50 mM potassium chloride, 2.5 mM magnesium chloride, 0.45% (v/v) NP-40, 0.45% (v/v) Tween-20, 50 µg/mL Proteinase K (Sigma)) followed by denaturing at 94°C for 15 minutes. HIV-1 proviral DNA was detected using the HIV-long terminal repeat (LTR) gag primer probe set as previously described [76]. The cell number was normalised to albumin as previously described [77]. The HIV-1 assay reaction contained Quantitative PCR SuperMix-UDG mastermix (Invitrogen), 300 nM forward primer, 300 nM reverse primer and 50 nM dual labelled probe and the albumin assay contained 150 nM dual labelled probe. After initial incubations of 50°C for 2 minutes and 94°C for 2 minutes, 45 cycles of amplification were carried out at 95°C for 15 seconds followed by 64°C for 45 seconds for the HIV-1 assay and 62°C for 45 seconds for the Albumin assay. The reaction run on the Corbett 3000 Rotor-Gene machine (Corbett Life Science; Sydney, NSW, Aus) and analysed using Corbett Rotor-Gene 6 software (version 6.0).

In addition, the expressions of selected genes were assessed using reverse transcribed total unamplified RNA as previously described [10]. The cDNA was subject to Q-PCR using published primers [10] with the addition of ICAM-1 primers: (Forward: CGTGGGGAGAAGGAGCTGAA, Reverse: CAGTGCGGCACGAGAAATTG, Sigma).

### Functional Studies

For the clustering assay, treated MDDC were co-cultured with autologous CD4^+^ T lymphocytes at a ratio of 1 DC∶5 T lymphocytes and incubated at 37°C for 45 minutes. Cells were subsequently stained for CD1a, CD3 and p24 for flow cytometric analysis as described above.

The proliferation was determined by carboxyfluroescein succinimidyl ester (CFSE) dilution. Briefly, allogeneic PBMCs were stained with 5 µM final concentration CSFE (Molecular Probes; Eugene, OR) for 10 minutes at 37°C, rescued with equal volume of 100% FBS and washed with RPMI+10% FBS. These PBMCs were added to the HIV-1_BaL_ infected MDDCs at a ratio of 1 MDDC∶10 PBMC and incubated at 37°C for 5 days. Cells were subsequently stained for CD3, CD4, CD8 and p24 for flow cytometric analysis as described above.

### Transfer of HIV-1 from DCs to T Lymphocytes

MDDCs infected with HIV-1_BaL_ (pellet or CD45^−^) for 48 hours were co-cultured with resting CD4^+^ T lymphocytes for a further 24, 48, 72, 96 and 120 hours at 37°C before staining with p24 antibody for flow cytometry as described above.

### Western Blot and Tangential Mass Spectrometry Analysis of Preparations

Viral, MV and parent cell preparations were lysed for 1 hour at 4°C in SDS lysis buffer (10 mM HEPES, 150 mM NaCl, 1% (v/v) Triton-X-100, 1 µg/mL protease inhibitor cocktail (Sigma) at pH of 7.5), followed by centrifugation for 10 minutes at 16,000×g at 4°C. The protein concentration was determined using the DC Protein Assay (Bio-Rad) according to the manufacturer's instructions. The protein concentration was determined using the DC Protein Assay (Bio-Rad) according to the manufacturer's instructions.

30 µg of protein prepared with 1× NUPAGE lithium dodecyl sulfate (LDS) sample buffer (Invitrogen) containing 400 mM dithiothreitol was boiled for 10 minutes before loaded into NUPAGE 4–12% polyacrylamide bis-tris gradient SDS-PAGE gels (Invitrogen) alongside a 10–250 kilo Dalton (kDa) molecular weight marker (Bio-Rad). Electrophoresis was run at 200 V for 50 minutes in NUPAGE 3-(N-morpholino)propanesulfonic acid (MOPS) SDS Running Buffer (Invitrogen). The proteins were transferred to a nitrocellulose membrane (Amersham Biosciences) in transfer buffer (250 mM Tris (pH 8.3), 1.92M Glycine and 0.05% (w/v) SDS) overnight at 55 mA. Non-specific binding sites were blocked by incubating for 1 hour in 250 mM Tris (pH 8), 1.4M NaCl and 30 mM KCl (pH 8) containing 20% (v/v) polyethylene glycol *tert*-octylphenyl ether and 5% skim milk). Blots were incubated with the primary antibodies in 1% skim milk solution followed by the appropriate peroxidase conjugated secondary antibody (Cell Signaling). Primary antibodies were: CD45 (clone 69/CD45, BD Transduction labs), exosome markers: Alix (3A9, Biolegend), CD9 (EPR2949, Abcam), CD81 (JS-81, BD Pharmingen), SMV markers: CD11a (HI111, Biolegend), CD40 (polyclonal, Abcam), AB markers: H2A (poly 6194) and H3 (poly6019, Biolegend) and maturation stimuli markers: CD40L (polyclonal, Abcam), TNF-α (Mab1, Biolegend), HSP60 (polyclonal), HSP70 (3C6), HSP90AB1 (4C10) and HSP90B1 (EPR3988, Origene). The membrane was developed using Western Lighting Enhanced Chemiluminescence Substrate Reagent and the Oxidising Reagent (Perkin Elmer; Glen Waverly, Vic, Aus).

For Mass Spectrometry, 30 µg of each sample was prepared in SDS lysis buffer, run on 4–12% bis-tris gradient SDS-PAGE gel and stained with Brilliant Blue G (Sigma).

The 1D SDS-Gel lanes were sliced into 31 1 mm×5 mm bands using a disposable grid cutter (The Gel Company, USA) and in-gel digested with trypsin using an automated liquid handling procedure with a TECAN Freedom Evo liquid handling system (Männedorf, Switzerland). The 31 fractions were pooled (2 gel fractions per pool) and analysed by LC-MS/MS in duplicate.

Peptide separation was performed on an Eksigent Nano 2D plus system (ABSciex, USA) employing splitless pumps enabled for nanoflow rates. RP-HPLC Trap and separation columns were prepared in-house (Supplementary data for extended Materials and Methods). For each LC run, sample was injected on the trap and washed for 10 minutes at 2.5 µL/min with loading buffer (2% v/v acetonitrile and 0.1% v/v Formic acid). Sample was separated by a linear gradient changing from 97% A (0.1% v/v formic acid in water) and 2% B (0.1% v/v formic acid in acetonitrile) to 40% A and 60% B in 60 minutes at 0.3 µL/min. The in-gel digested samples were analysed with a Thermo-Fisher LTQ-Velos Orbitrap. MS1 data were collected over the range of 300–2000 m/z in the Orbitrap set at resolution 30,000. FTMS preview scan and predictive automatic gain control (pAGC) were enabled. The full scan FTMS target ion volume was 1×10^6^ with a max fill time of 500 ms. MS2 data were collected in the LTQ-Velos with a target ion volume of 1×10^4^ and a max fill time of 100 ms. The 20 most intense peaks from a preview scan of each full Orbitrap scan were selected (with a selection window of 2.0 Da) for collision-induced dissociation (CID) with wide-band activation. Dynamic exclusion was enabled to exclude an observed precursor for 180 seconds after two observations. The dynamic exclusion list size was set at the maximum 500 and the exclusion width was set at ±5 ppm based on precursor mass. Monoisotopic precursor selection and charge state rejection were enabled to reject precursors with z = +1 or unassigned charge state.

Mass Spectrometry analysis involved converting Thermo .RAW files to mzXML format using MSConvert [78] and searched with X!Tandem [79] version 2010.10.01.1 (Supplementary data for extended Materials and Methods).

The identified peptides and their inferred proteins and spectral quantities are reported in the appended Microsoft Excel file (Mercier et al peptide-protein proteomic data.xlsx). The raw data for this project (103 Thermo .raw files, 53.5 Gb total) has been deposited in the Tranche proteomics raw data repository [80], [81] and may be downloaded from the Peptide Atlas data repository http://www.peptideatlas.org/PASS/PASS00251 or the ProteomeCommons.org Tranche repository using the following hash: “69iC+dKFUFu7JMcZxCSdI3cS0q37GPeG3yuWB2h32wnh27xemcTBoEY75tDGOGt0fGFes8Jyxf+ST5lv7Uz7lfUY5UQAA AAAAAAy3A =  = ”

### Supplementary Information

The raw data for this project (103 Thermo .raw files, 53.5 Gb total) has been deposited in the Tranche proteomics raw data repository [6], [7] and may be downloaded from the PeptideAtlas data repository

http://www.peptideatlas.org/PASS/PASS00251 or the ProteomeCommons.org

Tranche repository using the following hash:

“69iC+dKFUFu7JMcZxCSdI3cS0q37GPeG3yuWB2h32wnh 27xemcTBoEY75tDGOGt0fGFes8Jy 69iC+xf+ST5lv7Uz7lfUY 5UQAAAAAAAAy3A =  = ”

## Supporting Information

Figure S1 **SAMHD1 expression in MV treated maturing MDDCs.** MDDCs (0.5×10^6^/ml) were treated or mock treated with MVs from activated or non-activated primary CD4^+^ lymphocytes as in Figure 3D and lysates were processed for western blot as in Figure 7A, using antibodies to SAMHD1 (Abcam) and GAPDH.
(EPS)

Figure S2 **Recombinant HSP90α also induces maturation of MDDCs.** MDDCs (0.5×10^6^/ml) were treated with variable concentrations of recombinant HSP90α (similar to HSP90β) as in figure 7B and expression of maturation markers CD80, 83 and 86, MR and DC-SIGN measured by flow cytometry and expressed as log2fold change in MFI compared to mock treatment. (Mean +/− SE, n = 3, *p<0.05).
(EPS)
