# Correction: The Microvesicle Component of HIV-1 Inocula Modulates Dendritic Cell Infection and Maturation and Enhances Adhesion to and Activation of T Lymphocytes

**DOI:** 10.1371/annotation/059beb14-db84-4836-9fef-ec351946025a

**Published:** 2013-10-30

**Authors:** Sarah K. Mercier, Heather Donaghy, Rachel A. Botting, Stuart G. Turville, Andrew N. Harman, Najla Nasr, Hong Ji, Ulrike Kusebauch, Luis Mendoza, David Shteynberg, Kerrie Sandgren, Richard J. Simpson, Robert L. Moritz, Anthony L. Cunningham

The following grant information is missing from the Funding Statement:

US National Institutes for Health, National Cancer Institute for support (Grant # 1R03CA156667 to RM), the National Institute of General Medical Sciences (Grant #’s GM087221 and 2P50 GM076547 to RM) and the Luxembourg Centre for Systems Biomedicine and the University of Luxembourg (for support to RM). 

